# Bounce Forward: A School-Based Prevention Programme for Building Resilience in a Socioeconomically Disadvantaged Context

**DOI:** 10.3389/fpsyt.2020.599669

**Published:** 2021-01-14

**Authors:** Buket Kara, Rochelle Morris, Alice Brown, Pauline Wigglesworth, Joshua Kania, Angie Hart, Barbara Mezes, Josh Cameron, Suna Eryigit-Madzwamuse

**Affiliations:** ^1^Centre of Resilience for Social Justice, School of Health Sciences, University of Brighton, Brighton, United Kingdom; ^2^Blackpool Council, Blackpool, United Kingdom

**Keywords:** school based, prevention, disadvantaged (youth), mental health, youth, resilience (psychological), protective factors (resilience)

## Abstract

Socioeconomic status is a strong predictor of normative development and well-being in young people. It is well-known that growing up in a socioeconomically disadvantaged context may lead to negative outcomes, both in childhood and in adulthood. Early intervention and prevention programmes are crucial for building resilience and improving health, well-being and equity. Bounce Forward is a school-based prevention programme implemented in Blackpool, a town in the United Kingdom facing multiple challenges. It was part of a whole town resilience approach and nascent global social movement known as the “Resilience Revolution.” Between 2017 and 2019, the programme was delivered in all Year 5 classes at every primary school in Blackpool (*n*_*school*_ = 36), reaching out to 3,134 students (ages 9–10; 50.4% male). The programme aimed to increase resilience in young people by building knowledge and skills about mental health and resilience through 10 sessions. In the current study, we longitudinally examined a range of protective factors, which are relevant to young people's resilience, as well as their mental health outcomes at three time points: before they participated in Bounce Forward, at the end of the programme, and 3–5 months later, when they started Year 6. The current sample included 441 Year 5 students (54.2% male) from 11 primary schools in Blackpool. Nineteen teaching staff also participated in the study and provided qualitative data regarding the impact of the programme on their students. Results showed improvement in some areas of young people's resilience after taking part in Bounce Forward. We also identified gender differences in several protective factors, indicating that boys may need further support. Teaching staff highlighted improvements in various areas; and also observed that their students have been using the strategies that they learnt from the programme. Altogether, findings suggested that young people benefitted from Bounce Forward. The programme is sustainable, offering a free to download teacher resource pack that allows schools to self-deliver it.

## Introduction

Early adolescence is a critical period when young people develop knowledge and skills, attributes and abilities, and learn to manage emotions and relationships, which in turn shape their life in both adolescence and adulthood. It is also an important period for mental health, because research shows that half of all diagnoses of adult mental health disorders emerge in adolescence (1, 2), while the worldwide estimate of prevalence for diagnoses of mental health disorders in children and adolescents is 20% (3, 4). This percentage increases considerably when those with suboptimal mental health problems are also included. This means that at least one in every five children and adolescents experience a mental illness. Therefore, the promotion of child and adolescent mental health is crucial, not just to reduce societal and economical costs, but also to mitigate the inequalities gap between young people with social disadvantage and their more advantaged counterparts. It is our ethical responsibility to support young people who live with social disadvantages to reach their developmental potential (4). Early intervention and prevention programs hold major potential to prevent the onset of mental health difficulties and diagnoses of disorders, as well as to promote resilience in young people.

After exposure to challenging life experiences or adversity, particularly when those are chronic such as in the case of socioeconomic deprivation, young people may manifest distress responses, and in some cases, develop prolonged mental health problems (5, 6). Mental health challenges vary, but in general, girls tend to develop internalising problems (e.g., depression, anxiety, somatic complaints) while boys are more vulnerable to develop externalising problems (e.g., aggressive, behaviour, rule-breaking behaviour) (7, 8). However, not all young people develop mental health problems when they face stress or adversity, demonstrating a capacity for recovery and resilience (9). Resilience is described as a dynamic process that leads to “good outcomes in spite of serious threats to adaptation or development” (p. 228) (10). Research shows that young people's responses to adversity vary as a result of the interaction of specific individual and external factors, which are conceptualised and operationalised as risk factors and protective factors and shape the pathway to resilience (11). Among them, risk factors lead to a higher likelihood of a negative outcome, whereas protective factors are linked with the assets and resources that enhance positive and healthy development (12). Socioeconomic deprivation is one of the major risk factors that may cause non-optimal development and well-being in young people, which may cause or worsen many others such as parental distress, abuse and neglect, and lack of external support systems available to young people young people (6). However, a wide range of protective factors at the individual level (e.g., high self-esteem, good coping and problem-solving skills, empathy, future orientation, communication skills and prosocial behaviour), as well as at the wider context (e.g., supportive relationships with family members, friends and other people, opportunities for engagement within school and community) can help young people overcome the negative consequences of risk exposure (13, 14). Research further suggests that gender might play a role in accessing or using protective factors, as girls tend to report higher scores in various individual characteristics (e.g., empathy, problem solving) and better relationships in family, school, and community compared to boys (14).

More recent approaches to resilience incorporate a social justice lens, which recognise that inequality and social disadvantage contribute substantially to many adversities to which individuals, including young people, are exposed. Taking a public health perspective, this approach argues that interventions will not be successful or their impact will not be sustainable unless the structures that create the adversities are also challenged (15). Resilience, then, can be considered as “overcoming adversity, whilst also potentially changing, or even dramatically transforming (aspects of) that adversity” (p. 3) (15). Blackpool's test and learn pilot of the nascent global social movement known as the “Resilience Revolution” has been designed and led with this perspective.

Blackpool is a seaside town on the Lancashire coast in North West England. Despite being famous as a holiday destination, Blackpool is also one of the most socioeconomically deprived towns in England, which makes life challenging for young people to reach their potential. Thus, Blackpool has been selected as one of the six intervention areas across the country supported by the National Lottery funded programme, HeadStart, aiming to improve mental health and well-being of young people aged between 10 and 16 years and prevent mental health difficulties from developing (16). Blackpool's Resilience Revolution, which is the community-inspired name given to Blackpool HeadStart, is a whole town approach to addressing the mental health needs of young people in Blackpool piloting a nascent global social movement. It is a partnership of HeadStart Blackpool (led by Blackpool Council), the Centre of Resilience for Social Justice at the University of Brighton, and Boingboing Resilience Community Interest Company. The partnership uses Resilient Therapy (17) to develop new ways of working to support young people's mental health and well-being, with young people and their adult supporters involved as co-leaders. The overarching aim of the Resilience Revolution is to embed resilience-building approaches across whole areas of which Blackpool is the first, as well as to mobilise a social movement of collective action to tackle structural inequalities. In other words, the aim is to help individuals to “beat the odds” whilst also “changing the odds” for the whole community (p. 7) (15).

Blackpool's Resilience Revolution is led by the collaboration of individuals, organisations and services. One of these is Lancashire Mind, a charity aiming to make a difference to people's mental health. Bounce Forward was a universal resilience programme based on Resilient Therapy (17). The programme was co-developed by Blackpool HeadStart, Boingboing and Lancashire Mind, which offered a range of activities to Blackpool's young people. The aim of the programme was to increase young people's resilience by building knowledge and skills about mental health and resilience, so they would feel more equipped to respond when they face challenging life experiences. The programme and its delivery strategy were co-produced with the support of Blackpool HeadStart's Young People's Executive Group, a group of young people who were involved at every stage, from deciding the content and session planning, to designing the booklet for future use. The programme was managed by Lancashire Mind and implemented by their practitioners (known as Well-being Coaches), who had been trained in Resilient Therapy (17). The programme was delivered between 2017 and 2019 in all Year 5 classes at every primary school in Blackpool (n_school_ = 36), including three Special Educational Needs (SEN) schools. The Year 5 classes were targeted due to the programme's preventative approach, as these young people are edging toward transition to secondary school in Year 6. Transition from primary to secondary school is a critical period for all young people (18) but might be extra challenging for those who need additional support for any reason, including living in a socioeconomically challenging context.

The Bounce Forward sessions were underpinned by the Resilient Therapy approach, specifically through the Resilience Framework (17). The Resilience Framework includes 42 resilient moves under five components (i.e., basics, belonging, learning, coping, and core self) that are relevant to the resilience of young people (see Supplementary Material for the Framework). Each component offers simple, everyday actions—or resilient moves, which aim to help individuals become more resilient. The *Basics* covers the basic elements for a safe and healthy lifestyle, which are relevant to resilience, such as good-enough housing or exercise and fresh air. *Belonging* is tied to developing and keeping good relationships, knowing where you fit in the world, and focusing on good times and places. Thus, having a sense of belonging is essential for resilience, and resilient moves such as spending time with good people and in good places or having healthier relationships can help build resilience. *Learning* helps to develop new skills, be more organised, plan the future, and achieve goals, and corresponding resilient moves include making school work as well as possible or developing life skills. *Coping* refers to the strategies that help to manage tough situations and includes resilient moves such as being brave and remembering tomorrow is another day. The final component, *Core Self* , focuses on the thoughts and beliefs that build one's sense of self, and resilient moves for this component include knowing and understanding oneself and future orientation. Each session of Bounce Forward was closely linked to the aspects of the Resilience Framework, and highlighted strategies (i.e., resilient moves) to improve resilience and “bounce forward” through tough times (19). The delivery plan was also linked to different parts of the National Curriculum and Ofsted Requirements.

Bounce Forward was delivered as a ten-week programme, with a 1-h session per week. The sessions were designed in regard to meet specific needs of individuals. The delivery team were previously qualified teachers with knowledge and experience of inclusive teaching practises, behavioural management techniques, and neurodiversity inclusion, as well as SEND (special educational needs and disability) teaching practises. The session plans were shared in advance with the teaching and support staff in each school, so that the exercises could be amended to be accessible to all young people in the classroom. As comprehensively outlined in the resource pack (19), Bounce Forward started with an introductory session, introducing the programme and Resilience Revolution, as well as concepts such as resilience, well-being and their role in tough times. Sessions 2–9 covered components of the Resilience Framework and resilient moves. The sessions were highly interactive, involving various individual and group activities. The last session was planned and prepared by the young people as a school showcase, where they had a chance to embed their learning and display it to a school-wide audience, including other students and staff in the school, as well as parents/carers. School staff were also included in the programme through planning meetings, emails, and attending the Bounce Forward sessions. Therefore, staff gained knowledge about Bounce Forward, as well as the Resilience Revolution and its perspective on “beating the odds whilst also changing the odds” (p. 7) (15) to tackle structural inequalities to support mental health and well-being in young people.

The present research focused on the impact of the Bounce Forward programme on young people. Specifically, we longitudinally examined whether participating in Bounce Forward helped young people to improve their internal characteristics and external factors (i.e., protective factors), which are related to their resilience, and relatedly, whether there has been a change in the level of mental health difficulties that young people reported after they took part in the programme. For this, we collected data at three time points: before the programme, after the end of the programme, and a follow up, 3–5 months later. We predicted that, after taking part in the programme, young people would report higher levels of resilience and lower levels of mental health difficulties. The follow-up assessment occurred when young people were in Year 6, a critical and often challenging period as they are in the final preparations toward their transition to secondary school (18). Therefore, we expected that the strategies (e.g., resilient moves) that were taught in the programme would counteract the potential negative outcomes, and young people would report lower, or at least similar, mental health difficulties in Year 6. Drawing upon the previous literature, we also expected gender differences in reported mental health difficulties (7, 8) and protective factors (14). We predicted that, before the programme, girls would report higher emotional difficulties, whereas boys would report higher behavioural difficulties. We, then, expected that participating in the Bounce Forward programme would help girls to lower their emotional difficulties, and help boys to lower their behavioural difficulties. We also expected that girls would report higher levels of protective factors at the beginning of the programme compared to boys. Then, we explored the trajectories of change in the levels of protective factors reported by girls and boys. Finally, we explored young people's and school staff's perceptions of changes, if any, in young people's knowledge of and behaviour related to the subject of resilience. We expected to capture the positive impact of the programme in a classroom setting.

## Materials and Methods

### Participants

The study sample consisted of spring (January-March) and summer (April-July) term cohorts of Bounce Forward in the 2019 academic year. A total of 19 school staff (i.e., classroom teachers) and 441 Year 5 students (age 9–10; 54.2% male) attending 11 primary schools in Blackpool participated in the study. In the current sample, 92.5% of students' first language was English (compared to the national average of 78.8% for primary schools in 2019). During the spring term of 2019, 26.9% of the students were eligible for free school meals (compared to the national average of 17.1% for Year 5 classes in 2019), which is an indicator of low family income. Also, a further 14.6% of the students had a history of receiving free school meals for a period ranging from 1 to 15 school terms (out of 18 terms) before Spring 2019. During the spring term of 2019, 14.2% of the students were receiving special educational needs (SEN) support (compared to the national average of 15.1% for Year 5 classes in 2019), 0.7% were under an Education, Health and Care (EHC) plan (compared to the national average of 1.9% for Year 5 classes in 2019), and a further 9.6% had a history of SEN support for a period between 1 and 8 terms. In addition, four students had a history of being looked after, meaning they were under local authority care, for a period ranging 2–17 (out of 18) school terms.

The feedback from 2,795 young people, who took part in the Bounce Forward programme between September 2017 and December 2019, was also analysed in the current study.

### Procedure

Ethical approval was given by the Institutional Review Board of the University of Brighton (Life, Health, and Physical Sciences Cross School Research Ethics Committee). All participating young people provided verbal assent, and written consent was also provided from the young people's parents or carers. School staff who participated in the study also provided their written consent. Before data collection, all participants were informed about the confidentiality of their answers as well as their right to withdraw from the study.

Young people, including the ones with special needs, completed questionnaires on their own in a classroom with the assistance of Well-being Coaches and school staff present in the room. The completion time was ~30 min. The data was collected at three time points: before they participated in Bounce Forward (January-March 2019), at the end of the programme (April-July 2019), and 3–5 months later (October 2019), when they were in Year 6. Feedback forms were completed by young people and school staff after the end of the implementation.

### Measures

Young people were administered questionnaires to assess their perceptions of various protective factors that are relevant to their resilience, as well as the mental health difficulties that they experience. Table 1 includes the descriptive statistics and the internal consistency for the study variables. The internal consistency was calculated using Pearson's *r* for subscales with two items and Cronbach's α for subscales with three items or more. Young people and school staff were administered feedback forms to provide information about their perceptions of the programme. Finally, each young person had an income deprivation score based on their home postcode.

**Table 1:** Internal consistency and descriptive statistics for study variables (*N* = 441).

	**Baseline (T0)**					**After the programme (T1)**					**Follow up (T2)**				
**Variable (# of items)**	***IC***	***M***	***SD***	***Min***	***Max***	***IC***	***M***	***SD***	***Min***	***Max***	***IC***	***M***	***SD***	***Min***	***Max***
**Individual characteristics and external factors**															
Communication and cooperation (2)	0.42	3.95	0.76	1	5	0.54	3.99	0.79	1	5	0.55	3.93	0.80	1	5
Self-esteem (3)	0.70	3.80	0.85	1	5	0.76	3.90	0.88	1	5	0.77	3.83	0.90	1	5
Empathy (3)	0.69	4.07	0.96	1	5	0.73	4.09	0.95	1	5	0.74	4.04	0.96	1	5
Problem solving (3)	0.74	3.65	1.03	1	5	0.83	3.68	1.14	1	5	0.83	3.42	1.16	1	5
Goals and aspirations (2)	0.40	4.21	0.96	1	5	0.51	4.31	0.94	1	5	0.54	4.16	1.04	1	5
Family connexion (4)	0.52	4.33	0.58	2	5	0.64	4.37	0.62	2	5	0.73	4.38	0.66	1	5
School connexion (4)	0.76	4.20	0.79	1	5	0.81	4.21	0.80	1	5	0.83	4.12	0.83	1	5
Community connexion (4)	0.84	4.25	0.89	1	5	0.88	4.19	1.00	1	5	0.89	4.33	0.92	1	5
Participation in home and school (4)	0.69	3.35	0.87	1	5	0.70	3.40	0.87	1	5	0.76	3.27	0.93	1	5
Participation in community (2)	0.52	3.95	1.29	1	5	0.43	4.02	1.23	1	5	0.56	4.00	1.26	1	5
Peer support (13)	0.91	3.87	0.85	1	5	0.94	3.89	0.98	1	5	0.94	3.76	0.98	1	5
**Mental health difficulties**															
Emotional difficulties (10)	0.81	0.78	0.40	0	2	0.83	0.77	0.42	0	2	0.83	0.75	0.43	0	2
Behavioural difficulties (6)	0.83	0.65	0.47	0	2	0.87	0.74	0.51	0	2	0.84	0.58	0.46	0	2

*IC, Internal Consistency. The internal consistency was calculated using Pearson's r for subscales with two items and Cronbach's α for subscales with three items or more*.

#### Resilience

The Student Resilience Survey (20, 21) was used to measure young people's perceptions of individual characteristics and external protective factors in family, peer, and community contexts, which are relevant to their resilience. The psychometric studies show that the SRS has good reliability and validity (20, 21). The survey includes 47 items comprising 12 subscales: communication and cooperation (e.g., “I help other people”); self-esteem (e.g., “I can work out my problems”); empathy (e.g., “I feel bad when someone gets their feelings hurt”); problem solving (e.g., “I know where to go for help when I have a problem”); goals and aspirations (e.g., “I have goals and plans for future”); family connexion (e.g., “At home, there is an adult who listens to me when I have something to say”); school connexion (e.g., “At school, there is an adult who tells me when I do a good job”); community connexion (e.g., “Away from school, there is an adult who really cares about me”); participation in home and school life (e.g., “I help my family make decisions”); participation in community life (e.g., “I am a member of a club, sports team, or other group”); peer support (e.g., “There are students at my school who share things with me”); and pro-social friends (e.g., “My friends try to do what is right”). As our main focus was assessing protective factors that are available to young people, we did not use the pro-social peers subscale in the current study. Also, we used only two items (i.e., “I help other people”; “I enjoy working with other students”) of the communication and cooperation subscale, whereas the third item, “I stand up for myself” was disregarded to increase the reliability of the subscale. This also helped to prevent burdening young people with a long survey.

Young people rated the frequency of each item on a 5-point Likert-type scale ranging from 1 = *never* to 5 = *always*. For each subscale, we computed a final score by taking the average of responses given to the corresponding items.

#### Mental Health Difficulties

The Me & My Feelings scale (22) was used to assess young people's mental health in two broad domains: emotional difficulties and behavioural difficulties. The questionnaire has good reliability and validity (22) and comprises of 16 items, where 10 items assess emotional difficulties (e.g., “I cry a lot”), and 6 items assess behavioural difficulties (e.g., “I get very angry”). Young people rated the frequency of each item on a 3-point Likert-type scale (0 = *never*, 1 = sometimes, 2 = *always*). For each young person, emotional difficulties and behavioural difficulties scores were calculated by taking the average of responses given to the corresponding items.

#### Pupil Feedback Form

A pupil feedback form was developed to assess the perceptions of young people about the Bounce Forward programme. Young people first responded to two questions, including “Did you work on things that were important to you?” and “Overall, how did you feel about Bounce Forward?,” by rating on a 1–10 scale. Then, they responded to three open-ended questions: “The thing I liked best about Bounce Forward was: …,” “Bounce Forward could be made better by: ….,” and “How are you going to spread the message of the Resilience Revolution after Bounce Forward?”.

#### School Staff Feedback Form

A feedback form with four open-ended questions was developed to assess the perceptions of school staff (hereafter, teaching staff) about the programme. The questions included: “Would you be likely to recommend Bounce Forward to colleagues? Please explain.”, “Was Bounce Forward beneficial to your class? Please explain.”, “Do you think pupils increased their resilience after Bounce Forward and do you think they will use or apply any of the strategies they have learnt from the programme? Please explain.”, “Has there been any impact upon your own/your staff's knowledge and confidence around the subject of resilience? Do you feel confident in talking about resilience and the Bounce Forward programme in conversation with children, parents or colleagues?”.

#### Income Deprivation

The Income Deprivation Affecting Children Index (IDACI) (23) was used to assess the income deprivation rank based on the postcode where young people resided. Income deprivation is considered when people are either out-of-work or in work but have low earning. With this respect, the IDACI shows the proportion of all children aged from 0 to 15 living in income-deprived families in a given area in 2019. In our sample, the IDACI ranged between 1 and 10 with a mean of 3.14 (*SD* = 1.86), where 1 is the most deprived 10%.

### Data Analysis

Quantitative data analysis was performed using IBM SPSS Statistics 26.0 for Windows. Prior to analysis, study variables were explored for accuracy of data entry and missing values. Overall, the rate of missing values ranged between 0.2 and 1.4% for baseline and T1, and there were no missing values for T2 assessment. We replaced the missing values in our data for each subscale separately by using Expectation-Maximisation algorithm. The data was also explored for meeting the assumptions of variance analysis.

To address our research questions, we performed a two-way mixed-design analysis of covariance (ANCOVA). ANCOVA was chosen to examine the changes in young people's scores before and after taking part in the Bounce Forward programme, while reducing within-group variance and eliminating potential confounds such as income deprivation and special needs. Accordingly, we examined the difference in resilience and mental health difficulties scores in regards to gender of young people and across the time points: Before the programme (baseline), end of the programme (T1), and follow-up 3–5 months later (T2). The SEN support status (0 = none, 1 = on support) of young people at the time of implementation and income deprivation (i.e., IDACI scores) were included in the analysis as covariates. Pairwise *post-hoc* comparisons were conducted using Bonferroni correction.

Finally, the qualitative data collected from young people and teaching staff were analysed using NVivo 12. A predominantly inductive approach to thematic analysis (24) was adopted to identify the themes that emerged from the data.

## Results

As presented in Table 1, young people reported moderate to high levels of protective factors, both for individual characteristics and external factors. Also, they reported low levels of emotional and behavioural disorders.

### Resilience

ANCOVA results, controlling for SEN support status and income deprivation, revealed a significant interaction of time and gender in communication and cooperation of young people, while gender and time were not significant (for statistics, see Table 2).

**Table 2:** ANCOVA statistics for study variables controlling for income deprivation and SEN support status (*N* = 441).

**Outcome**	**Source**	***F***	***df***	***p***	***$\eta_{p}^{2}$***
**Individual characteristics and external factors**					
Communication and cooperation	Time	1.05	2	0.35	0.00
	Gender	0.63	1	0.43	0.00
	Time*Gender	5.27	2	0.01	0.01
Self-esteem	Time	0.13	2	0.88	0.00
	Gender	0.82	1	0.37	0.00
	Time*Gender	2.10	2	0.12	0.00
Empathy	Time	1.23	2	0.37	0.00
	Gender	21.54	1	0.00	0.05
	Time*Gender	2.44	2	0.09	0.00
Problem solving	Time	10.44	2	0.00	0.02
	Gender	4.72	1	0.03	0.01
	Time*Gender	0.69	2	0.50	0.00
Goals and aspirations	Time	3.47	2	0.03	0.01
	Gender	0.59	1	0.55	0.00
	Time*Gender	0.00	2	0.96	0.00
Family connexion	Time	0.09	2	0.91	0.00
	Gender	3.04	1	0.08	0.01
	Time*Gender	1.42	2	0.23	0.00
School connexion	Time	0.88	2	0.41	0.00
	Gender	4.59	1	0.03	0.01
	Time*Gender	0.11	2	0.90	0.00
Community connexion	Time	1.46	2	0.23	0.00
	Gender	7.55	1	0.01	0.02
	Time*Gender	5.27	2	0.02	0.01
Participation in home and school	Time	0.80	2	0.45	0.00
	Gender	35.44	1	0.00	0.08
	Time*Gender	0.62	2	0.54	0.01
Participation in community	Time	1.17	2	0.31	0.00
	Gender	13.47	1	0.00	0.03
	Time*Gender	1.59	2	0.21	0.00
Peer support	Time	2.68	2	0.07	0.01
	Gender	16.77	1	0.00	0.04
	Time*Gender	0.08	2	0.92	0.00
**Mental health difficulties**					
Emotional difficulties	Time	0.28	2	0.75	0.00
	Gender	1.55	1	0.20	0.00
	Time*Gender	0.34	2	0.72	0.00
Behavioural difficulties	Time	15.19	2	0.00	0.03
	Gender	26.66	1	0.00	0.06
	Time*Gender	0.74	2	0.48	0.00

**Figure 1:**
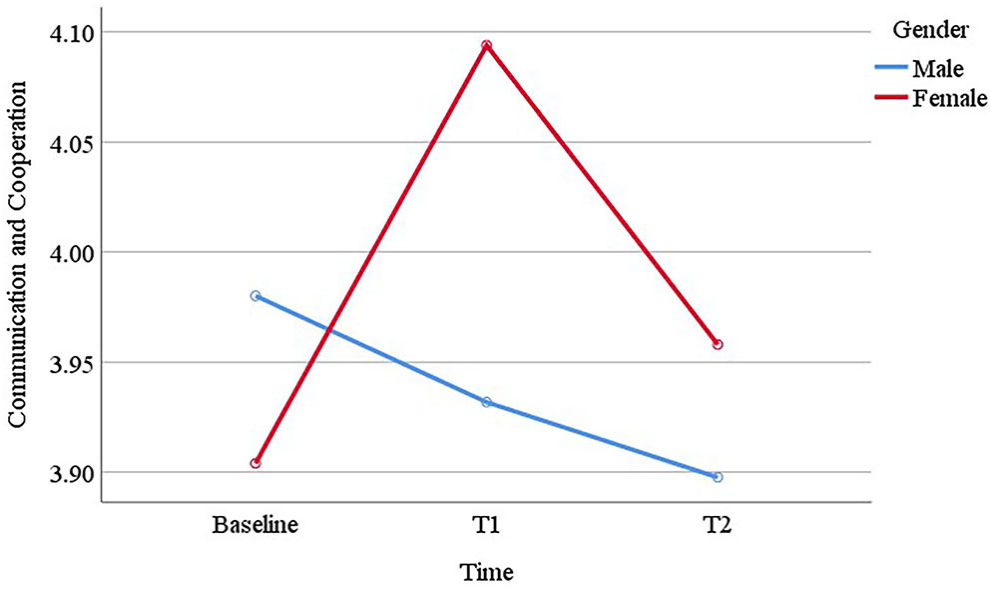
The interaction of time and gender in young people's communication and cooperation scores.

**Figure 2:**
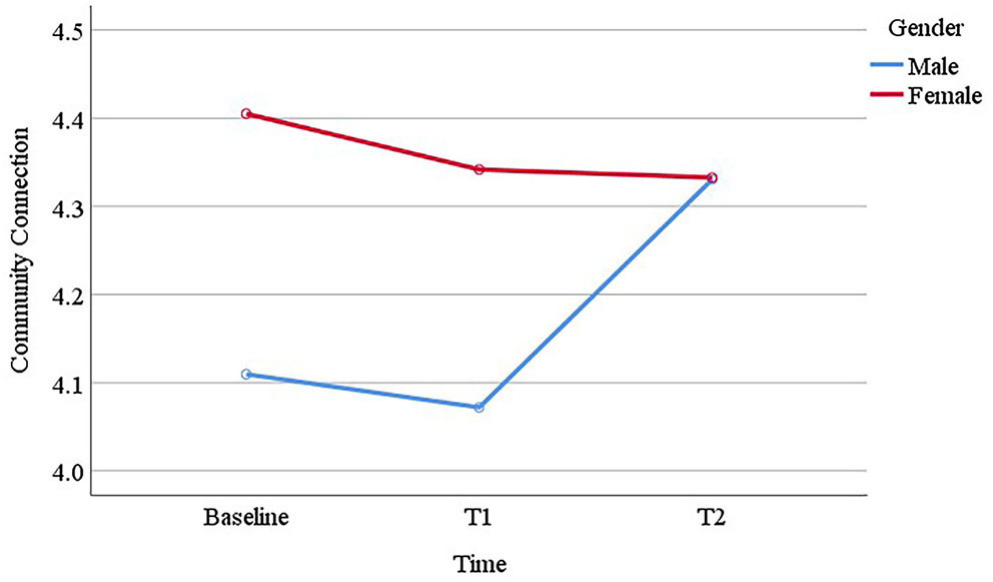
The interaction of time and gender in young people's community connection scores.

Boys reported similar communication and cooperation scores across baseline (*M* = 3.98, *SE* = 0.05), T1 (*M* = 3.93, *SE* = 0.05), and T2 (*M* = 3.90, *SE* = 0.05), whereas girls reported higher scores at T1 (*M* = 4.09, *SE* = 0.05) compared to baseline (*M* = 3.90, *SE* = 0.05), which decreased at T2 (*M* = 3.96, *SE* = 0.05; see Figure 1). For self-esteem, time, gender and their interaction were not significant. However, a significant gender difference was observed for empathy scores, where girls (*M* = 4.25, *SE* = 0.05) scored higher than boys (*M* = 3.91, *SE* = 0.05). Time and interaction of time and gender did not differentiate young people in empathy. Problem solving was another protective factor where we observed significant effect of time and gender, whereas their interaction was not significant. *Post-hoc* analysis revealed that baseline (*M* = 3.65, *SE* = 0.05) and T1 (*M* = 3.71, *SE* = 0.05) scores were similar, but there was a significant decrease from baseline to T2 (*M* = 3.43, *SE* = 0.06; *p* < 0.001) and from T1 to T2 (*p* < 0.001). Also, girls (*M* = 3.69, *SE* = 0.06) reported higher problem-solving skills than boys (*M* = 3.50, *SE* = 0.06). For the next protective factor, “goals and aspirations,” analysis revealed significant changes across time. Pairwise comparisons showed a marginal increase from baseline (*M* = 4.21, *SE* = 0.05) to T1 (*M* = 4.32, *SE* = 0.04; *p* = 0.06), which decreased from T1 to T2 (*M* = 4.15, *SE* = 0.05; *p* = 0.001). Gender and interaction of time and gender did not differentiate young people in their goals and aspirations. For family connexion, the analysis revealed a marginal gender difference, where girls (*M* = 4.41, *SE* = 0.04) scored slightly higher than boys (*M* = 4.32, *SE* = 0.03), but the results were non-significant for time and the interaction of time and gender. Another gender difference was found for school connexion, where girls (*M* = 4.25, *SE* = 0.05) reported significantly higher school connexion in comparison to boys (*M* = 4.12, *SE* = 0.04). Time and the interaction of time and gender did not differentiate young people in their school connexion. For community connexion, a significant gender difference was revealed where girls' (*M* = 4.36, *SE* = 0.05) scores were overall higher than boys (*M* = 4.17, *SE* = 0.05). The interaction of time and gender was also significant (see Figure 2). Boys reported similar community connexion across baseline (*M* = 4.11, *SE* = 0.06) and T1 (*M* = 4.07, *SE* = 0.06), which increased at T2 (*M* = 4.33, *SE* = 0.06); whereas girls reported similar scores across baseline (*M* = 4.41, *SE* = 0.06), T1 (*M* = 4.34, *SE* = 0.07) and T2 (*M* = 4.33, *SE* = 0.07). For participation in home and school, gender significantly differentiated girls (*M* = 3.57, *SE* = 0.05) and boys (*M* = 3.15, *SE* = 0.05), but time and the interaction of time and gender were not significant. Similar results were observed for participation in community, where girls (*M* = 4.18, *SE* = 0.07) reported significantly higher level of participation in community than boys (*M* = 3.84, *SE* = 0.07), but the results for time and the interaction of time and gender were non-significant. Finally, gender also significantly differentiated girls (*M* = 4.01, *SE* = 0.06) and boys (*M* = 3.70, *SE* = 0.05) in their peer support. Time was found marginally significant, where T2 score (*M* = 3.77, *SE* = 0.05) was slightly lower than baseline (*M* = 3.89, *SE* = 0.04; *p* = 0.02) and T1 (*M* = 3.91, *SE* = 0.05; *p* = 0.001). The interaction of time and gender was not significant.

### Mental Health Difficulties

As for mental health difficulties (for statistics, see Table 2), results showed that time, gender or their interaction did not play a significant role in young people's emotional difficulties. However, for behavioural difficulties, we observed significant results for time and gender. Pairwise comparisons revealed that young people reported higher behavioural difficulties at T1 compared to baseline (*p* < 0.001); whereas at T2, their scores were significantly lower than both baseline (*p* < 0.001) and T1 (*p* < 0.001). A significant gender difference was also revealed where girls' (*M* = 0.55, *SE* = 0.03) scores were overall lower than boys (*M* = 0.75, *SE* = 0.03). The interaction of time and gender, however, was found to be not significant.

### Feedback From Young People

The proportion of young people who considered they worked on things that were important to them during Bounce Forward sessions was 82.8% (rated 6 or above on a 1–10 scale; *M* = 8.00, *SD* = 2.32, range = 1–10). Overall, 89.4% of young people reported that they enjoyed taking part in Bounce Forward (rated 6 or above on a 1–10 scale; *M* = 8.68, *SD* = 2.16, range = 1–10).

Young people's qualitative feedback revealed the aspects of the programme that they liked the most, their recommendations to improve the programme, and how they would share their learning and knowledge about resilience with others. The themes emerged from young people's responses included:

#### Having Fun While Learning About Resilience

Session and activity names such as “Positivity glasses” and “Kid president” (see the resource pack for details) were frequently mentioned (around 20 and 10% of young people, respectively), together with “fun” (around 9%). Many young people found the activities helpful, and at the same time enjoyed the process of learning about resilience. Some young people considered positivity glasses to be a tool that they would continue to use to feel positive when facing challenges.

“*I thought that making the ‘positivity glasses' was the best because they now make me feel positive about what I need them for and also ‘kid president' because he is funny what he does.”*
“*[…] we were with friends. The things we did are awesome because we learnt to be more resilient and to always have fun. #alwayshavefun”*
“*I enjoyed doing all of the fun activities and I have learnt to be more resilient.”*

Around 10% of the young people expressed a general appreciation of the whole process, and how all the activities were helpful and enjoyable.

“*All the videos and how helpful all the staff were.”*
“*That it was an amazing way to make me more resilient. Not one of the lessons has been pointless. They have all meant something to me. My favourite thing to do was the glasses and cup activity.”*

Only 5 young people out of 2,795 responded by saying “*nothing*,” indicating that they did not enjoy or benefit from the activities and sessions.

#### Feeling Listened to/Empowered

Many young people (around 10%) expressed that they felt listened to and empowered, which helped them to become more creative, make plans about their own future, and improve their relationships with peers and teachers.

“*[…] the people really helped and understood everything and listened to me, the activities were FUN, thank you.”*
“*I loved that when I had my hand up, they never cut me off. They always listened!”*

#### Improving Bounce Forward

Around 15% of the young people suggested that Bounce Forward does not need any change.

“*It doesn't need to be improved, it's already a really good experience for children.”*

Around 15% of the young people suggested having more frequent and longer sessions, more creative and fun activities, and more games.

“*Bounce Forward could be more than an hour because they have helped me become more confident in tough times.”*
“*Making more time, […to] make more things that we think we are good at. For example I like crafting, so we [could] do things like that.”*
“*More videos, more teamwork activities, more ‘games' that build resilience”*

Around 10 young people also suggested going on a trip would help them with aspects of their resilience.

“*Going on a trip to somewhere–it would make us trust other people”*

#### Spreading the Resilience Revolution

Young people responded that they planned to spread the message of the Resilience Revolution after Bounce Forward by being more resilient (around 15%). Around 30 young people also expressed that they considered using their “positivity glasses” for this.

“*I'm going to spread the message by going to show people how to be resilient”*
“*Show people my glasses, my book and tell them how important it is.”*

Around 15% of the young people stated that they planned to spread Resilience Revolution to their siblings/family members (around 15%). Many young people (around 15%) planned to talk about the Resilience Revolution with other people or advise/support others. Some young people envisaged using social media, posters, or videos to do this.

“*(I will) tell my mum and dad or family members”*
“*I am going to spread the message by telling all of my friends that are not in this school and they can tell other people”*
“*Facetime my friends about bounce forward”*
“*Making YouTube video on it.”*
“*Creating posters and stick them around school.”*

### Feedback From School Staff

All staff responded that they would recommend Bounce Forward to their colleagues. The staff described the implementation of the programme as engaging, enjoyable, reflective, interesting, relevant, informative, and creative. A small number of staff suggested the programme could be implemented more effectively through a higher number of sessions, longer sessions, and more practical activities such as role-playing.

#### Impact on Young People

Staff highlighted the positive impact of the programme on their pupils, including key themes such as:

##### Greater Understanding of Resilience and Practising Resilient Moves

Many staff reflected that participating in Bounce Forward improved their students' understanding of resilience and taught them strategies (e.g., resilient moves) that they started practising.

“*Children can be seen and heard talking about and sharing resilient moves and practising them in class and the playground.”*
“*Children use the strategies taught (e.g., positivity glasses) in class.”*
“*(The programme) taught them how to concentrate on the positives and that by looking at the framework, they could identify what ways they already use to be resilient and what new resilient steps they could try.”*
“*Yes, I think they are a lot more likely to recognise the resilient moves they do and are more aware of it and what they can do to put these in place.”*
“*They understand that there are ways they can help themselves be happy and healthy by exercise, healthy eating, brushing their teeth, etc. And spending time with good people in good places. Many of our students see school as their ‘safe place' [where they can carry out Resilience Framework [moves] such as ‘transport', ‘healthy eating', ‘having a laugh', ‘problem solving' and ‘spending time with good people in good places', but it has also taught them how to transfer this into other environments, including ones where they may not feel as comfortable. I think this gives them an opportunity to use these skills as important life skills for now and the future.”*

A small number of staff considered that their students might be using or applying resilience strategies that they have learned during the programme, but either they did not have enough time to observe that, or not all children were engaged with the strategies. Nevertheless, the staff were optimistic.

“*Hopefully the children will use the strategies consistently and with growing independence, only time will tell!”*
“*Some children still need to mature into resilience - however, we now have a good grounding to refer to with those who struggle. This is so useful!”*
“*Hopefully! They are able to speak about it but aren't necessarily applying it to their own lives.”*

##### Improved Relationships and Behaviour

Some staff reflected that taking part in Bounce Forward help their students improve their relationships and behaviour. A small number of them also observed an increase in young people's empathy and supportive behaviour toward one another in particular.

“*Relationships between children improved.”*
“*Reminding the children to look at the resilient framework has had an impact especially on behaviour and relationships.”*
“*Some children struggling with attendance showing improvement with coming into school.”*
“*Children more able to understand how to manage relationship issues.”*
“*The children are more aware of the feelings of one another and are more likely to support each other.”*
“*Perhaps some impact on relationships with each other through a better understanding and empathy.”*

##### Resilience for Schoolwork and Learning

Some staff observed that, after taking part in Bounce Forward, their students have been using strategies that they learnt from the programme in their schoolwork and learning.

“*When tackling new and challenging work in mathematics the children have been showing more resilience.”*
“*Some children have been wearing their ‘positivity glasses' in class to focus and when their resilience has needed to be concentrated on.”*
“*Children are already using strategies learnt and the programme has enabled them to think a lot more before acting. Resilience towards their work has had a remarked improvement for some children. For others it has cemented the resilience already built up.”*
“*Pupils talk about the strategies they have learnt. Showcase assembly planning shows the depth of enthusiasm and learning that has taken place.”*

It is worth noting that, although acknowledging some improvements, a small number of staff also reported that they did not observe improvements in all areas or a tangible impact.

“*It allowed some of the quieter, shyer members of the class to express their feelings and concerns. I haven't noticed a specific impact upon behaviour and attendance.”*
“*[…]Pupils [are] able to discuss and explain resilience, the moves and identify strengths/areas for development.”*

##### Awareness of Emotions, Strengths and Weakness, and Resilience

Some staff reflected that taking part in Bounce Forward helped young people improve their understanding of emotions, their strengths and weaknesses, and resilience.

“*Gives the children a good grounding in exploring their emotions and dealing with difficult times.”*
“*Fantastic for children to recognise their strengths/weaknesses and how to become resilient to life's stumbling blocks.”*

##### Improved Ability to Express Emotions

After participating in the Bounce Forward programme, some staff observed that their students had not only understood their emotions more fully, but also became more able to communicate how they are feeling.

“*Children are happy to talk about things that are bothering them. It has created an ethos of discussion.”*
“*(The programme was beneficial to) encourage talking and keeping open.”*
“*They are more likely to discuss how they are feeling and have the language/vocabulary to enable them to explain.”*

##### Problem Solving and Decision-Making

A small number of staff reflected that participating in Bounce Forward contributed to their students' problem solving and decision-making skills.

“*(The programme is) very helpful for encouraging the children to help solve problems and make their own decisions.”*
“*[…] the children are more able to suggest ideas to solve (or resolve) a problem - with just a few prompts.”*
“*The children now try to mediate and offer advice when situations occur.”*
“*Children have shown resilience by referring to strategies learnt in Bounce Forward sessions to help solve problems.”*

#### Impact on Teaching Staff

Staff highlighted the positive impact of the programme not only for young people but for themselves too. The themes emerged from their responses, included:

##### Greater Knowledge and Confidence in the Subject of Resilience

One of the clear benefits from the programme was linked to increasing staff knowledge and confidence around resilience. The majority of staff reflected that Bounce Forward helped them to develop this. Staff commented that, after Bounce Forward, they felt more confident to talk about resilience and the Bounce Forward programme with different stakeholders such as students, parents, and colleagues.

“*I have learnt that resilience takes many forms and the children can demonstrate it in a number of ways.”*
“*The resilience framework is easy to follow and shows that everyone can use resilient moves.”*
“*I would feel confident talking about their framework/Bounce Forward with parents or colleagues.”*
“*I feel totally confident in delivering the subject of resilience as well as talking about it to other stakeholders.”*

##### Improved Confidence to Identify and Help Students Become More Resilient

A small number of staff responded that they became more aware of students who are less resilient than others and they felt more confident to use the techniques that they learnt from Bounce Forward to approach these young people and help them become more resilient.

“*[I have become] more aware of children who are less resilient than others and how to approach this using Bounce Forward techniques.”*
“*I have found the programme helpful and I have gained confidence in helping the children to choose to be more resilient.”*

Some staff reported using references to specific sessions, specific activities, or specific resilient moves to help young people to be resilient.

“*When there have been fallouts between friends I have encouraged ‘bouncing back' and giving the children more ownership over solving problems.”*
“*They are definitely more aware of what resilience means and I am therefore able to refer to it and to the resilient moves when necessary.”*

One staff also commented shifting their perspective on the value of Bounce Forward as a school-based programme.

“*I was unsure about this programme when I first heard due to the fact that I felt life experiences should be learned ‘naturally'. However, not only do I now see how valuable this programme is but I would also have been interested in finding more information about it to help others.”*

## Discussion

Adversity in early adolescence may cause mental health difficulties in young people. These challenges might be at individual level (e.g., transition from primary school to secondary school), at family level (e.g., parental unemployment), or at a wider environmental level (e.g., socioeconomic deprivation in the community). Young people living in Blackpool are under the risk of exposure to many challenges, and the cumulative effect of these risks may compromise their well-being to a greater extent (12, 25). Bounce Forward is a school-based prevention programme implemented in Blackpool to build resilience in young people and equip them to overcome challenging life experiences. The current study aimed to research the impact of Bounce Forward with a group of young people and teaching staff, and the results partly confirmed our predictions.

Our findings suggested that participating in Bounce Forward helped improve a number of protective factors that are relevant to young people's resilience in Year 5 and 6. Notably, after participation in the programme, young people reported higher levels of goals and aspirations. Having plans and aspirations for the future helps young people to become more resilient when times are tough (13, 17). However, this increase was not carried over in time to Year 6. A similar trend was observed for communication and cooperation, a quality that is shown to help overcome adversity (13). In our sample, girls reported higher scores for communication and cooperation after the end of the programme, which then declined in Year 6. For problem solving, another protective factor which buffers the effects of adversity (13, 17), we also observed a decrease in young people's scores in Year 6. One explanation for this trend might be that the positive effect of the programme might simply end by Year 6. However, a more plausible explanation might be that getting closer to transition to a secondary school in Year 6 led to lower scores and indeed scores may have been even lower without the buffering effect of participation in Bounce Forward. These findings were in line with the literature. Early adolescence is a period of change in young people's social context, including transition from primary school to secondary school. It is normative during this period for young people to experience decreased self-esteem, reduction in their social support, and more mental health problems (26–28). Therefore, identifying no significant differences might be an indication of positive outcomes. This was the case in our sample. We found that many protective factors (e.g., connexion to and participation in family and school, peer support) that we assessed did not decrease in Year 6. On the contrary, we observed that boys scored higher community connexion in Year 6. This might be related to participating in Bounce Forward, where boys, who overall scored lower at the baseline, possibly improved their relationships with adults outside of family and school.

Findings also highlighted significant gender differences in various individual characteristics and external factors that are relevant to young people's resilience. In line with the literature (14), overall, girls reported higher scores in empathy and problem solving, as well as connexion to and participation in family, school and community, and peer support. This may indicate a higher level of socio-emotional development in girls compared to boys, and that boys may need further support in these areas. Strategies to provide this may include screening for boys that would need extra help supporting their socio-emotional development and providing individualised support on a one-to-one basis. Remarkably, our findings indicated similar levels of self-esteem in girls and boys both in Year 5 and Year 6. This was in contradiction to studies which indicate that self-esteem significantly decreases in girls while increasing in boys during early adolescence (29, 30), and to studies which report an overall decrease of self-esteem in young people as they approach transition to secondary school (25, 30). This may suggest that participating in Bounce Forward counteracted with this normative developmental trend and helped young people, particularly girls, to maintain their self-esteem level while nearing transition to secondary school.

Child and adolescent development research suggests that, with puberty, girls are more vulnerable to experience internalising problems (e.g., depression, anxiety, somatic complaints) whereas boys are more likely to develop externalising problems (e.g., aggressive behaviour, rule-breaking behaviour) (7, 8). In line with this literature, we found that boys reported higher levels of behavioural difficulties compared to girls in our sample. Contrary to our expectations, however, young people reported significantly higher behavioural difficulties at the end of the programme compared to baseline. It is possible to explain this finding as arising from young people gaining increased awareness of their behavioural issues as a result of what they learnt and reflected on from the programme. Supporting this argument, we later observed that behavioural difficulties scores reported in Year 6 were even lower than the baseline scores. Because the transition period is associated with higher problem behaviours in young people (18, 26, 31) the decrease in behavioural difficulties we observed in Year 6 is critical, suggesting that participating in Bounce Forward helped young people to overcome negative consequences of transition. For emotional difficulties, in contrast to the literature (7, 8), we found that girls reported similar scores to boys. This might be because girls, in our sample, scored high in many individual characteristics and external factors that function as protective, decreasing the likelihood of mental health difficulties. Relatedly, we also expected to find that participating in Bounce Forward would help young people to decrease the emotional difficulties that they experienced. Findings did not support this prediction, as young people's scores were similar at baseline, the end of the programme, and in Year 6. Nevertheless, this may still indicate a positive impact of the programme, because the literature suggested a significant increase in emotional difficulties closer to transition period (18, 26, 31), and in our sample, young people's emotional difficulty scores did not increase in Year 6.

Feedback from young people and teaching staff also helped evaluate the impact of Bounce Forward on both young people and school staff. Notably, the majority of young people reported that they addressed issues that were important to them during the Bounce Forward sessions and that they enjoyed the programme. Many young people reported experiencing fun while learning about resilience and resilient moves. They considered that it was also the session structure and activities which helped them improve their resilience. Examples of this included feeling listened to and empowered, becoming more creative, making plans about their own future, and improving relationships with peers and teachers. Young people were happy to be a part of the Resilience Revolution, and they planned to spread its message by being more resilient, sharing their knowledge of resilience with family, peers and other people, and advising or supporting others when times are tough. Similarly, the teaching staff were willing to recommend the programme to others as they were satisfied with both the content and impact of the programme. Furthermore, the teaching staff considered that their students benefitted from the programme in numerous ways, such as developing greater understanding of resilience, emotions, and strengths and weakness. Staff observed that their students were using strategies (e.g., resilient moves) that they learnt from the programme to improve their relationships and behaviour (e.g., attendance), that they were more willing to talk about their emotions, and that they demonstrated improved empathy and supportive behaviour. Students also improved their problem-solving skills and resilience for schoolwork and for learning new and challenging topics, which contributed to their school performance. School staff considered that they too developed greater knowledge of and confidence with the subject of resilience, which helped them to identify students who were less resilient than others, and to support them to become more resilient.

It is also important to underline that, in our sample, young people's scores were high for protective factors and low for mental health difficulties even before the implementation of the programme. This could have resulted from the indirect impact of the overall whole town Resilience Revolution pilot in Blackpool. Previous programmes implemented in schools might have increased the likelihood of building resilience knowledge and have created awareness in families and at schools. Throughout the programme, young people were encouraged to spread the message of resilience to as many people as they could inside and outside of school. Similarly, showcases that young people prepared and presented at the end of Bounce Forward provided a space where they shared their knowledge gained from the programme with other students and staff in the school and their families. It should also be noted that Bounce Forward, and in general the Resilience Revolution pilot, aimed to involve school staff in the programmes too. Therefore, being part of the Resilience Revolution might have changed the school climate to support young people's mental health and well-being in advance or parallel to Bounce Forward implementation *per se*.

Bounce Forward was implemented as a part of Blackpool's Resilience Revolution pilot, which adopts a whole-town approach to embed resilience-building approaches across the town. The Resilience Revolution supports young people's mental health and well-being by mobilising collective action to tackle structural inequalities and social disadvantage. It is recognised that these inequalities and social disadvantage create adversities for many young people and their families. Without challenging or transforming these adversities, interventions may not be successful or sustainable (15). Bounce Forward was designed and implemented with this perspective, and introduced young people to strategies (i.e., resilient moves) (17, 19) to improve aspects of their resilience and bounce forward when times are tough. The current study showed that Bounce Forward had a positive impact on both young people and school staff in terms of building knowledge of resilience and mental health. Our findings revealed that young people were using resilient moves, which in turn either helped them to improve certain areas related to their resilience and behaviour, reducing the likelihood of behavioural difficulties, or which prevented the normative decline in areas of their resilience. Even though Bounce Forward focused more on equipping young people to “beat the odds,” rather than “change the odds,” the programme still contributed to the Resilience Revolution—both directly with its positive impact on young people and teaching staff, and indirectly by increasing school staff's awareness of Resilient Therapy tools to tackle structural inequalities and social disadvantage at schools.

### Strengths and Limitations

Findings of the current study must be considered and interpreted with respect to its strong and weak methodological features. To start with its strengths, the current study presented longitudinal data which allowed us to examine trends or trajectories of change, as well as dynamics of individual behaviour, while giving insight into the potential causal processes. Another strength of the current study was that we used both qualitative and quantitative data from multiple respondents (i.e., young people and teaching staff). This helped us to explore the impact of the Bounce Forward programme more in depth and breadth.

In terms of limitations, as Bounce Forward was a universal programme implemented in every school, our sample did not include a control group. This prevented us from eliminating alternative explanations of our findings. We, therefore, used the normative trends in the literature for early adolescence as a reference to evaluate our findings. Another limitation could be that our findings regarding the level of mental health difficulties were based on young people's self-reports. However, literature points out important discrepancies in emotional and behavioural problems of children reported by parents, teachers and children (32, 33). This cross-informant discrepancy suggests that information collected from a single informant might not be sufficient for a comprehensive assessment of children's emotional and behavioural problems. It could also be argued that the time span of the longitudinal assessment was limited, allowing us to examine the trajectories only for a short time. Another weakness of the current study is that, even though Bounce Forward was developed and administered within a whole-town approach, the potential impact of wider system on the delivery and impact of Bounce Forward was not fully explored within the scope of this study. Future studies with a more integrated study design would be required for such evaluation of wider system. Furthermore, collecting data from multiple respondents, including young people, families and other stakeholders, regarding structural inequities would provide valuable evidence to explore the role of the Resilience Revolution and the whole-town approach.

### Conclusion

Grounded in the Resilience Framework (17), Bounce Forward is a school-based prevention programme implemented as a part of Blackpool's Resilience Revolution using a whole-town approach. The current study showed that the programme efficiently introduced young people to resilient moves, and it had a positive impact on both young people and school staff. At the wider level, by building resilience in the young population and introducing schools to resilience through a social justice lens, the programme also has potential to continue contributing to Blackpool's community. Bounce Forward is sustainable, and can be self-delivered by schools it using the resource pack created for teachers (19).

## Data Availability Statement

The original contributions presented in the study are included in the article/Supplementary Materials, further inquiries can be directed to the corresponding author/s.

## Ethics Statement

The studies involving human participants were reviewed and approved by Life, Health, and Physical Sciences Cross School Research Ethics Committee, University of Brighton. Written informed consent to participate in this study was provided by the participants' legal guardian/next of kin.

## Author Contributions

BK designed the study, prepared the data for analysis, conducted statistical analysis, interpreted data, wrote the first draft and revised the versions of the manuscript, and approved the final version. RM acquired and managed quantitative data collection, prepared the data for analysis, contributed to the interpretation of data from a local authority practise perspective, critically revised the manuscript, and approved the final version. AB acquired and managed qualitative data collection, prepared the data for analysis, contributed to the interpretation of data from a local authority practice perspective, critically revised the manuscript, and approved the final version. PW contributed to the interpretation of data from a local authority practise perspective, critically revised the manuscript, and approved the final version. JK contributed to the design and delivery of the programme, critically revised the manuscript, and approved the final version. AH contributed to the interpretation of data from a social justice oriented resilience perspective, critically revised the manuscript, and approved the final version. BM contributed to the design and delivery of the programme, obtained the ethical approval, managed data collection, critically revised the manuscript, and approved the final version. JC planned, obtained funding, and contributed to the interpretation of the qualitative data, critically revised the manuscript, and approved the final version. SE-M planned, obtained funding and designed the overall longitudinal study, conceptualised, supervised and coordinated the current study, interpreted data, critically revised versions of the manuscript, and approved the final version. Blackpool's pilot of the Resilience Revolution, obtained HeadStart funding for the implementation funding, designed and delivered the programme, revised the versions of the manuscript, and approved the final version. All authors contributed to the article and approved the submitted version.

## Conflict of Interest

The authors declare that the research was conducted in the absence of any commercial or financial relationships that could be construed as a potential conflict of interest.

## References

[1] Belfer ML. Child and adolescent mental disorders: the magnitude of the problem across the globe. J Child Psychol Psychiatry. (2008) 49:226–36. 10.1111/j.1469-7610.2007.01855.x 18221350

[2] Kessler RC Berglund P Demler O Jin R Walters EE. Lifetime prevalence and age-of-onset distributions of DSM-IV disorders in the National comorbidity survey replication. Arch Gen Psychiatry. (2005) 62:593–602. 10.1001/archpsyc.62.6.593 15939837

[3] World Health Organization Atlas: Child and Adolescent Mental Health Resources. Geneva: World Health Organization (2005).

[4] Kieling C Baker-Henningham H Belfer M Conti G Ertem I Omigbodun O. Child and adolescent mental health worldwide: evidence for action. Lancet. (2011) 378:1515–25. 10.1016/S0140-6736(11)60827-1 22008427

[5] Aber JL Bennett NG Conley DC Li J. The effects of poverty on child health and development. Annu Rev Public Health. (1997) 18:463–83. 10.1146/annurev.publhealth.18.1.463 9143727

[6] Bradley RH Corwyn RF. Socioeconomic status and child development. Annu Rev Psychol. (2002) 53:371–99. 10.1146/annurev.psych.53.100901.135233 11752490

[7] Rescorla L Achenbach T Ivanova M Dumenci L Almqvist F Bilenberg N Behavioral and emotional problems reported by parents of children ages 6 to 16 in 31 societies. J Emotion Behav Disord. (2007) 15:130–42. 10.1177/10634266070150030101

[8] Zahn-Waxler C Shirtcliff EA Marceau K. Disorders of childhood and adolescence: gender and psychopathology. Annu Rev Clin Psychol. (2008) 4:275–303. 10.1146/annurev.clinpsy.3.022806.091358 18370618

[9] Garmezy N Masten AS Tellegen A. The study of stress and competence in children: a building block for developmental psychopathology. Child Develop. (1984) 1:97–111. 10.2307/1129837 6705637

[10] Masten AS. Ordinary magic: resilience processes in development. Am Psychologist. (2001) 56:227–38. 10.1037/0003-066X.56.3.227 11315249

[11] Masten AS Narayan AJ. Child development in the context of disaster, war, and terrorism: pathways of risk and resilience. Annu Rev Psychol. (2012) 63:227–57. 10.1146/annurev-psych-120710-100356 21943168 PMC5858878

[12] Fergus S Zimmerman MA. Adolescent resilience: a framework for understanding healthy development in the face of risk. Annu Rev Public Health. (2005) 26:399–419. 10.1146/annurev.publhealth.26.021304.144357 15760295

[13] O'Connell ME Boat T Warner KE. Preventing Mental, Emotional, and Behavioral Disorders among Young People: Progress and Possibilities. Washington, DC: National Academies Press (2009). 20662125

[14] Sun J Stewart D Age and gender effects on resilience in children and adolescents. Int J Mental Health Promot. (2007) 9:16–25. 10.1080/14623730.2007.9721845

[15] Hart A Gagnon E Eryigit-Madzwamuse S Cameron J Aranda K Rathbone A Uniting resilience research and practice with an inequalities approach. Sage Open. (2016) 6:1–13. 10.1177/2158244016682477

[16] National Lottery Community Fund Headstart. (2020). Available online at: https://www.tnlcommunityfund.org.uk/funding/strategic-investments/headstart#section-1 (accessed August 20, 2020).

[17] Hart A Blincow D Thomas H Resilient Therapy: Working with Children and Families. Hove: Routledge (2007).

[18] Coffey A Relationships: the key to successful transition from primary to secondary school? Improving Schools. (2013) 16:261–71. 10.1177/1365480213505181

[19] Boingboing Bunce Forward - Teacher Pack 2019. (2020). Available online at: https://www.boingboing.org.uk/bounce-forward (accessed August 20, 2020).

[20] Lereya ST Humphrey N Patalay P Wolpert M Böhnke JR Macdougall A. The student resilience survey: psychometric validation and associations with mental health. Child Adolesc Psychiatry Ment Health. (2016) 10:44. 10.1186/s13034-016-0132-5 27822304 PMC5093941

[21] Sun J Stewart D Development of population-based resilience measures in the primary school setting. Health Educ. (2007) 107:575–99. 10.1108/09654280710827957

[22] Deighton J Tymms P Vostanis P Belsky J Fonagy P Brown A. The development of a school-based measure of child mental health. J Psychoeduc Assess. (2013) 31:247–57. 10.1177/0734282912465570 25076806 PMC4107815

[23] Ministry of Housing Communities and Local Government English Indices of Deprivation 2019. (2019). Available online at: https://www.gov.uk/government/statistics/english-indices-of-deprivation-2019 (accessed August 20, 2020).

[24] Braun V Clarke V Using thematic analysis in psychology. Qualit Res Psychol. (2006) 3:77–101. 10.1191/1478088706qp063oa

[25] Rutter M. Psychosocial resilience and protective mechanisms. Am J Orthopsychiatry. (1987) 57:316–31. 10.1111/j.1939-0025.1987.tb03541.x 3303954

[26] Evans D Borriello GA Field AP. A review of the academic and psychological impact of the transition to secondary education. Front Psychol. (2018) 9:1482. 10.3389/fpsyg.2018.01482 30210385 PMC6123573

[27] Cantin S Boivin M Change and stability in children's social network and self-perceptions during transition from elementary to junior high school. Int J Behav Dev. (2004) 28:561–70. 10.1080/01650250444000289

[28] Crockett LJ Petersen AC Graber JA Schulenberg JE Ebata A School transitions and adjustment during early adolescence. J Early Adolesc. (1989) 9:181–210. 10.1177/0272431689093002

[29] Watkins D Dong Q Xia Y. Age and gender differences in the self-esteem of Chinese children. J Soc Psychol. (1997) 137:374–9. 10.1080/00224549709595448 9200974

[30] Wigfield A Eccles JS Mac Iver D Reuman DA Midgley C Transitions during early adolescence: changes in children's domain-specific self-perceptions and general self-esteem across the transition to junior high school. Dev Psychol. (1991) 27:552–65. 10.1037/0012-1649.27.4.552

[31] Elias MJ Transitioning to middle school. Educ Digest. (2002) 67:41–3.

[32] Rescorla LA Ginzburg S Achenbach TM Ivanova MY Almqvist F Begovac I. Cross-informant agreement between parent-reported and adolescent self-reported problems in 25 societies. J Clin Child Adolesc Psychol. (2013) 42:262–73. 10.1080/15374416.2012.717870 23009025

[33] Rescorla LA Bochicchio L Achenbach TM Ivanova MY Almqvist F Begovac I. Parent–teacher agreement on children's problems in 21 societies. J Clin Child Adolesc Psychol. (2014) 43:627–42. 10.1080/15374416.2014.900719 24787452

