# Supplementary material for: Bounce Forward: A School-Based Prevention Programme for Building Resilience in a Socioeconomically Disadvantaged Context
Source: Front Psychiatry. 2021 Jan 14;11:599669. doi: 10.3389/fpsyt.2020.599669 (PMC7840841; doi:10.3389/fpsyt.2020.599669)
Supplement: Supplementary file 1 [file Data_Sheet_1.docx]

Supplementary Material


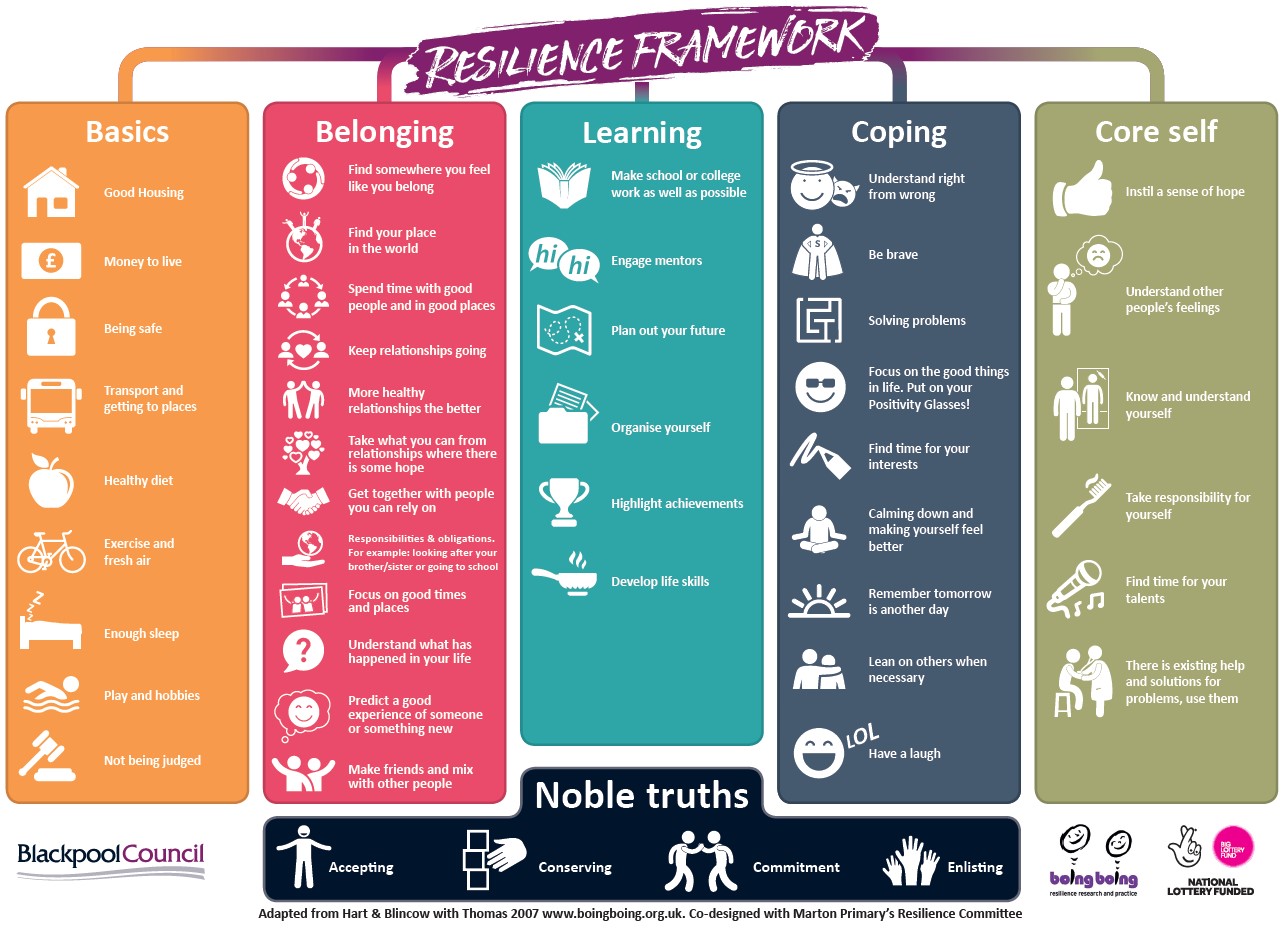


**Supplementary Figure 1.** Resilience Framework, adapted from ‘Hart A, Blincow D, Thomas H. Resilient Therapy: Working with children and families. Routledge (2007)’ and co-designed with the Resilience Committee at Marton Primary School, Blackpool. Other formats and languages are accessible from: <https://www.boingboing.org.uk/resilience/resilient-therapy-resilience-framework/>

.
